# Tumor-Induced Osteomalacia and the Importance of Plasma Fibroblast Growth Factor 23 as an Indicator: Diagnostic Delay Leads to a Suicide Attempt

**DOI:** 10.1177/2324709619895162

**Published:** 2019-12-18

**Authors:** LaRae Seemann, Sandeep Anand Padala, Azeem Mohammed, Nardos Belayneh

**Affiliations:** 1 Augusta University Medical Center, Medical College of Georgia, Augusta, GA, USA; 2 Eastside Premier, Covington, GA, USA

**Keywords:** tumor-induced osteomalacia, osteomalacia, hypophosphatemia, renal phosphate wasting, paraneoplastic syndromes, phosphaturic mesenchymal tumor, fibroblast growth factor 23, FGF23, octreotide scintigraphy

## Abstract

Tumor-induced osteomalacia is a rare hypophosphatemic disease caused by unregulated production of fibroblast growth factor 23 by a tumor, thereby inducing renal phosphate wasting and inhibiting appropriate increase of calcitriol production. Symptoms of tumor-induced osteomalacia, including muscle weakness, bone pain, and pathologic fractures, are nonspecific and warrant further workup. We report the case of a 50-year-old African American female with no known psychiatric illness who was admitted after a failed suicide attempt provoked by severe bone pain. She had been treated for fibromyalgia and hypophosphatemic rickets at other facilities with no improvement. The findings of profound renal phosphate wasting initiated further evaluation, which revealed an elevated fibroblast growth factor 23 level and a right proximal fibular mesenchymal tumor on octreotide scintigraphy. Magnetic resonance imaging confirmed the findings of a solid intramuscular tumor corresponding to the octreotide avid lesion. After wide excision of the tumor, serum phosphate and parathyroid hormone levels began to normalize. This case highlights the importance of extensively investigating the cause of bone pain, weakness, and fatigue in patients without a family history of hypophosphatemia or bone disorders. The aforementioned symptoms may precede recurrent pathological fractures, and a thorough workup ensures that a diagnosis of tumor is not delayed or overlooked, as tumor resection confers a favorable prognosis and dramatic increase in the quality of life for patients.

## Introduction

Tumor-induced osteomalacia (TIO) is a rare paraneoplastic syndrome caused by unregulated production of fibroblast growth factor 23 (FGF23) by a mesenchymal tumor. Excessive circulating FGF23 acts as a phosphaturic hormone, inducing renal phosphate wasting and inhibiting an appropriate increase in circulating calcitriol, which ultimately leads to hypophosphatemia.^1,2^ Chronic hypophosphatemia results in inadequate bone mineralization and presents clinically as osteomalacia. Symptoms may be nonspecific with an insidious onset, and include, but are not limited to, fatigue, proximal muscle weakness, bone pain, and gait disturbances.^3,4^ We report the case of a 50-year-old African American female with no known psychiatric illness who was admitted after a failed suicide attempt provoked by severe bone pain.

## Case Presentation

A 50-year-old African American female with no known history of psychiatric illness was admitted to the psychiatry service after a suicide attempt that she attributed to unbearable arthralgias and recurrent fractures for the last 18 months. The patient had been brought by Emergency Medical Service after an attempt to stab herself with a butcher knife because the pain had become so severe over the previous several days. Outside of our facility, the patient had been treated with duloxetine for fibromyalgia, then with calcium carbonate and sodium phosphate for presumed hypophosphatemic rickets with no improvement. We were consulted for evaluation of incidental hypophosphatemia. The patient complained of severe generalized bone pain, fatigue, and recent rib fracture. She was taking diclofenac sodium 35 mg, fentanyl transdermal patch 12 µg, and tizanidine 2 mg for generalized bone pain with no relief. Vital signs were normal, and a musculoskeletal examination was negative for tenderness, hyperemia, and joint swelling. All other physical findings were unremarkable. Laboratory tests (Table 1) indicated serum phosphorous 1.2 mg/dL, serum parathyroid hormone (PTH) 183 ng/mL, serum 1,25(OH)D 16 pg/mL, urine phosphate 700 mg/dL, and fractional excretion of phosphate 50% (>5% in the setting of hypophosphatemia indicates renal phosphate wasting).

**Table 1.** Laboratory Findings on Admission.

CMP	
Phosphorus	1.2 mg/dL (2.5-4.8)
Magnesium	2.1 mEq/L (1.5-2.5)
Alkaline phosphate	304 IU/L (44-147)
1,25(OH)D total	26
1,25(OH)D	16 pg/mL
PTH	183 ng/mL (14-64)
Serum Cr	0.6 mg/dL
24-Hour urine	
Volume	1225 mL
Phosphate	59.8 mg/dL
Cr	54 mg/dL
Calculated total UCr	648 mg/24 hours
Total urine phosphate	700 mg/day
FE PO_4_^−^	50%

Abbreviations: CMP, comprehensive metabolic panel; PTH, parathyroid hormone; Cr, creatinine.

**Figure 1.**
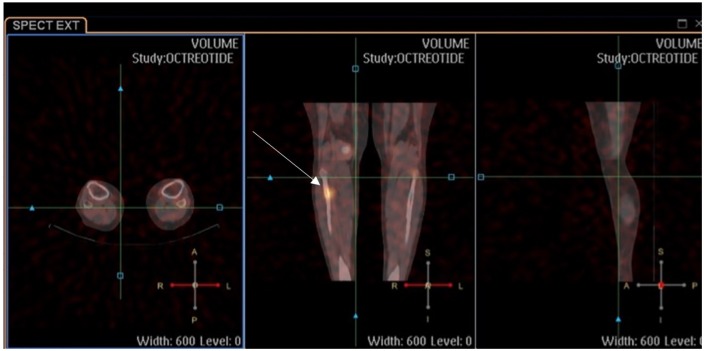
Intense focally increased uptake within a 0.5-cm lytic bony lesion in the anteromedial proximal right fibular cortex (arrow), consistent with the culprit mesenchymal tumor producing tumor induced osteomalacia.

**Figure 2.**
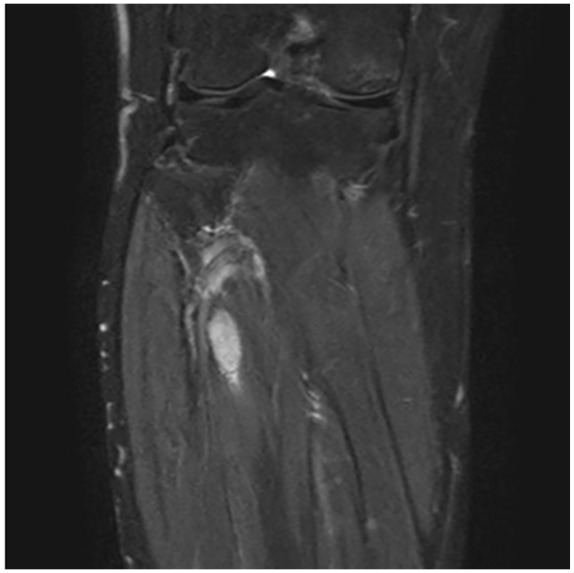
Magnetic resonance imaging of solid intramuscular tumor corresponding to octreotide avid lesion.

**Figure 3.**
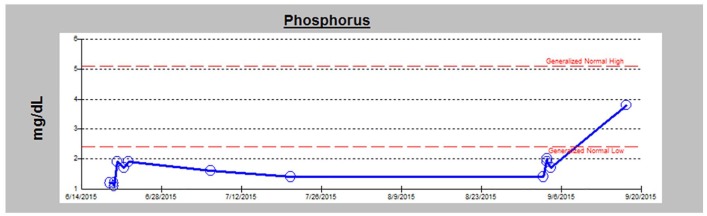
Serum phosphorus trend over 3 months, with an increase seen post tumor excision.

Given the profound renal phosphate wasting, we checked a FGF23 level that returned high at 364 RU/mL. The elevated FGF23 prompted a search for a latent neoplasm causing TIO. Functional imaging was ordered, and an octreotide scan showed a right proximal fibular mesenchymal tumor (Figure 1). Anatomical imaging (magnetic resonance imaging) confirmed the findings (Figure 2). Orthopedics was consulted for wide excision of the tumor, which involved both bone and muscle tissue. After excision of the tumor, serum phosphate and PTH levels normalized to 50 ng/mL and 4 mg/dL, respectively (Figure 3). Patient reported satisfaction with significant improvement in her bone pain and also complete resolution of her psychiatric symptoms. She was able to discontinue use of duloxetine, diclofenac, fentanyl transdermal patch, and tizanidine.

## Discussion

Hypophosphatemia can be induced by decreased intestinal absorption, acute movement of extracellular phosphate into the cells, renal replacement therapy, or increased urinary phosphate excretion. A 24-hour urine phosphate excretion helps differentiate between appropriately low renal phosphate excretion and renal phosphate wasting. Renal phosphate wasting suggests that the cause of hypophosphatemia is hyperparathyroidism or primary renal phosphate wasting. Primary forms of renal phosphate wasting include several genetic forms resulting from different gene mutations, TIO, and Fanconi syndrome. Rarely, renal phosphate wasting is observed in patients with fibrous dysplasia and McCune-Albright syndrome. Workup of hypophosphatemia should include TIO in the differential. TIO should be differentiated from inherited hypophosphatemic disorders, but if there is a positive family history, TIO cannot be automatically excluded. The degree of FGF23 elevation may be useful in differentiating inherited or acquired hypophosphatemia from TIO. In a cross-sectional study in Japan, TIO patients showed higher serum levels of FGF23 than patients with either Fanconi syndrome or vitamin D deficiency.^5^

FGF23 acts as a phosphaturic factor and suppresses 1α-hydroxylase activity in the kidney. It is expressed in osteocytes in bone, pericyte-like cells surrounding the venous sinuses in bone marrow, and in the thymus and lymph nodes.^6^ Perhaps most important, osteoblast-mediated bone formation is driven by a bone-kidney axis that is largely regulated by FGF23 expression. FGF23-mediated receptor activation requires a single-pass transmembrane protein called Klotho as a cofactor. Deficiency of klotho also appears to reduce osteoblastic population and interfere with bone mineralization.^7^ Together, the FGF receptor–Klotho complex inhibits sodium-dependent phosphate uptake and 1α-hydroxylase activity in the proximal tubule of the kidney, leading to hypophosphatemia and inappropriate low 1,25(OH)_2_D.^8^ In contrast, a deficiency of either FGF23 or klotho results in the opposite phenotype of hyperphosphatemia and elevated production of 1, 25(OH)_2_D, further confirming the role of FGF23 in regulation of serum phosphate and 1,25(OH)_2_D levels.

In the diagnosis and treatment of hypophosphatemic disorders, FGF23 is an essential indicator. There are 3 ELISA assays for measuring circulating levels of FGF23, one of which is more sensitive for use on patients with TIO.^6^ The Kainos intact assay should be selected over the immunotopics C-terminal and intact assays for TIO patients.

Despite the biochemical hallmarks of the disorder, TIO is often missed due to lack of knowledge or failure to investigate serum phosphate and FGF23 levels, and patients frequently present physically debilitated and depressed from chronic pain. Although there is limited literature describing the specific relationship between TIO and depression, the general relationship between chronic pain and depression is well described. Research suggests comorbidity is attributable to shared neurotransmitters, neuromodulators, cytokines, and receptors.^9,10^ This suggests that there may also be dysregulated neurobiology explaining depression in the setting of TIO. Bone pain is the most commonly reported symptom of TIO and tends to start in the lower limb.^11^ Symptoms may precede recurring pathologic fractures, the cause of which is often misdiagnosed or undiagnosed. One retrospective study reported the initial misdiagnosis rate for TIO as 95.1%, with the most common misdiagnoses being intervertebral disc herniation, spondylarthritis, and osteoporosis.^11^ Because of the insidious and sometimes clinically ambiguous nature of TIO, biochemical findings play a critical role in the diagnosis. If hypophosphatemia is discovered, TIO should be considered. The typical biochemical abnormalities include low serum phosphate, normal to low calcium, normal PTH, low or inappropriately normal 1,25-dihydroxy vitamin D (1,25(OH_2_D)), normal 25-hydroxyvitamin D (25(OH)D), elevated alkaline phosphatase, elevated serum FGF23, and increased phosphate excretion in the urine with tubular maximum phosphate reabsorption per glomerular filtration rate reduction.^3,4^ FGF23 is an essential indicator for not only the diagnosis, but also the management of TIO.

If a tumor is successfully localized, complete tumor excision is the most effective approach. In a study of 40 patients, Sun et al^12^ concluded that 80% of the cohort was treated successfully through tumor resection, with 88% achieving normal serum phosphorus during the study. In patients who refuse surgical intervention, computed tomography–guided radiofrequency ablation may be an effective therapy.^13^ FGF23 can be used to monitor the disease course postsurgically to ensure levels return to normal.^2^ Most often, biochemical abnormalities resolve with complete tumor resection and are paralleled by significant clinical improvement.

## Conclusion

Tumor-induced osteomalacia is a rare paraneoplastic syndrome that must be included in the differential diagnosis of a patient presenting with hypophosphatemia and bone pain. The diagnosis is often delayed for years because of its nonspecific nature, but early diagnosis and tumor excision dramatically improve quality of life.
